# A Rare Case of a Giant Bladder Stone Associated With Post-obstructive Renal Failure Managed by Open Cystolithotomy

**DOI:** 10.7759/cureus.39718

**Published:** 2023-05-30

**Authors:** Janie Hu, Alexander T Phan, Debra Craig

**Affiliations:** 1 Internal Medicine, Arrowhead Regional Medical Center, Colton, USA

**Keywords:** internal medicine, nephrology, post-obstructive aki, giant bladder stone, urolithiasis

## Abstract

Urinary tract stones are found in many locations, such as in the kidney or ureter, and, less commonly, in the bladder. Bladder stones are solid calculi that are usually composed of calcified material, most commonly uric acid, and typically weigh less than 100 g. There is a higher prevalence of bladder stones in males than in females, which can be explained by the pathophysiology of how these stones are formed. Namely, bladder stones tend to form secondary to urinary stasis, such as in the setting of benign prostatic hyperplasia (BPH). However, bladder stones can form in otherwise healthy individuals without anatomic defects (e.g., urethral strictures) or urinary tract infections (UTIs). Foley catheters or any foreign bodies in the bladder can predispose to stone formation. Renal calculi, most commonly calcium oxalate or calcium phosphate in composition, can also travel through the ureter and get trapped in the bladder. The most significant risk factors for bladder stones include the presence of BPH and UTIs, both of which favor the development of additional layers of stone material. In exceptionally rare cases, bladder stones measure more than 10 cm in diameter and weigh more than 100 g. These entities have been referred to as giant bladder stones within the limited literature. Minimal data exist on the etiology, epidemiology, composition, and pathophysiology of giant bladder stones. We present the case of a 75-year-old male with a giant bladder stone composed of 100% carbonate apatite, measuring 10 cm × 6 cm and weighing 210 g.

## Introduction

Urinary tract stones, also referred to as urolithiasis, can be found in several locations, including the kidneys, ureters, urethra, and bladder [1]. The prevalence of urolithiasis in economically developed countries has been reported to be between 4% and 20% [2,3]. Vesicolithiasis is a subset of urolithiasis that refers to stones that form in the urinary bladder and the primary cause is urinary stasis, as in the case of benign prostatic hyperplasia and neurogenic bladder. Bladder stones may develop as a result of small kidney stones that pass through the ureters and remain in the bladder, foreign material retained in the bladder, radiation therapy, schistosomiasis, urethral strictures, urethral diverticula, and bladder augmentation surgery [4]. Vesicolithiasis is less common than other etiologies of urolithiasis, making up approximately 5% of urolithiasis.

The most common stone composition of bladder calculi is uric acid (50%), with other common compositions including calcium oxalate, calcium phosphate, ammonium urate, cysteine, and struvite. Bladder stones may present asymptomatically, though when symptomatic, they may cause terminal hematuria, suprapubic pain, impaired bladder voiding, and dysuria [4]. Bladder stones weighing over 100 g are called giant bladder stones and their occurrence is exceedingly rare [1,2]. Giant bladder stones may also be associated with the development of urinary tract infections, acute kidney injury, and bilateral hydronephrosis [1,2,5,6].

We report the case of a 75-year-old male patient who presented with complaints of abdominal pain and was found to have severe post-obstructive acute kidney injury. Imaging studies demonstrated a large bladder stone. The patient eventually received an open cystolithotomy because of the massive size of the stone.

## Case presentation

A 75-year-old male with a past medical history of hypertension and recurrent nephrolithiasis presented to our emergency department due to lower abdominal pain associated with a lack of urinary catheter drainage. Notably, he was admitted to an outside hospital one month prior for a urinary tract infection and bilateral hydronephrosis due to obstructive nephrolithiasis that was managed with antibiotics and urinary catheter placement. The patient reported that his abdominal pain was also associated with back pain, rated 5 out of 10 in severity, and was exacerbated by movement. He denied nausea, vomiting, diarrhea, fevers, chills, night sweats, or gross hematuria. Initial vital signs included a temperature of 97.9°F, blood pressure of 142/82 mmHg, pulse rate of 85 beats/minute, respiratory rate of 15 breaths/minute, and oxygen saturation of 99% on ambient air. Physical examination revealed an ill-appearing male as stated age, bilateral costovertebral angle tenderness, diffuse tenderness to lower abdominal palpation, abdominal distension, and poor dentition. Initial laboratory investigations demonstrated leukocytosis, normocytic anemia, elevated serum creatinine, elevated parathyroid hormone, and decreased vitamin D (Table 1). A urinalysis revealed increased leukocyte esterase, positive nitrites, mild proteinuria, pyuria, increased erythrocytes, and hematuria (Table 2).

**Table 1:** Initial serum laboratory studies demonstrating leukocytosis, normocytic anemia, and elevated serum creatinine.

Laboratory test	Reference values	Measured values
White blood cells (cells/μL)	4,300–11,100	11,700
Hemoglobin (g/dL)	11.5–15.5	10.2
Hematocrit (%)	36–46	31
Platelet (cells/μL)	120,000–360,000	266,000
Mean corpuscular volume (fL)	80–100	91
Sodium (mEq/L)	135–148	138
Potassium (mEq/L)	3.5–5.5	3.7
Chloride (mEq/L)	98–110	104
Bicarbonate (mmol/L)	24–34	25
Blood urea nitrogen (mg/dL)	8–20	13
Creatinine (mg/dL)	0.5–1.5	1.39
Calcium (mg/dL)	8.5–10.5	10.4
Phosphorous (mg/dL)	2.4–4.4	3.3
Albumin (g/dL)	3.5–4.9	3.7
25-Hydroxycholecalciferol (ng/mL)	30–100	17.8
Parathyroid hormone (pg/mL)	15–65	92.01

**Table 2:** Urine laboratory studies. hpf: high-power field

Laboratory test	Reference values	Measured values
Urine color	Yellow, dark yellow, green, straw	Yellow
Urine clarity	Clear	Cloudy
pH	5–8	5.5
Leukocyte esterase	Negative	3+
Nitrite	Negative	Positive
Protein	Negative	1+
Glucose	Negative	Negative
Bilirubin	Negative	Negative
Ketones	Negative	Negative
Blood	Negative	3+
Specific gravity	1.003–1.035	1.014
White blood cells	0–5/hpf	Packed
Red blood cells	0–4/hpf	Packed
Bacteria	Negative/hpf	1+
Squamous epithelial cells	None/hpf	None

**Figure 1:**
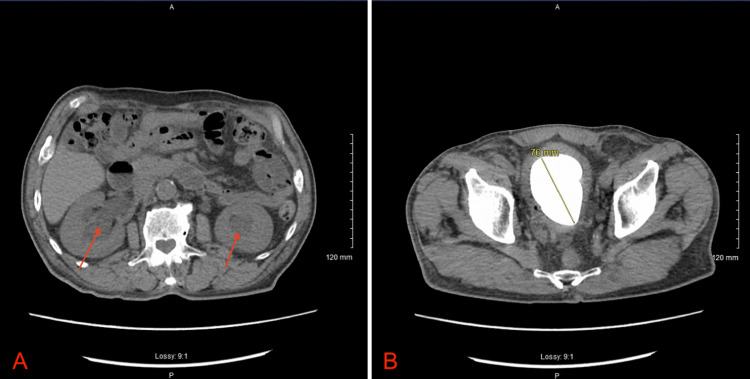
(A) Axial section of computed tomography of the abdomen and pelvis demonstrating bilateral renal hydronephrosis (red arrows). (B) Axial section of computed tomography of the abdomen and pelvis demonstrating a 7.6 cm bladder stone.

**Figure 2:**
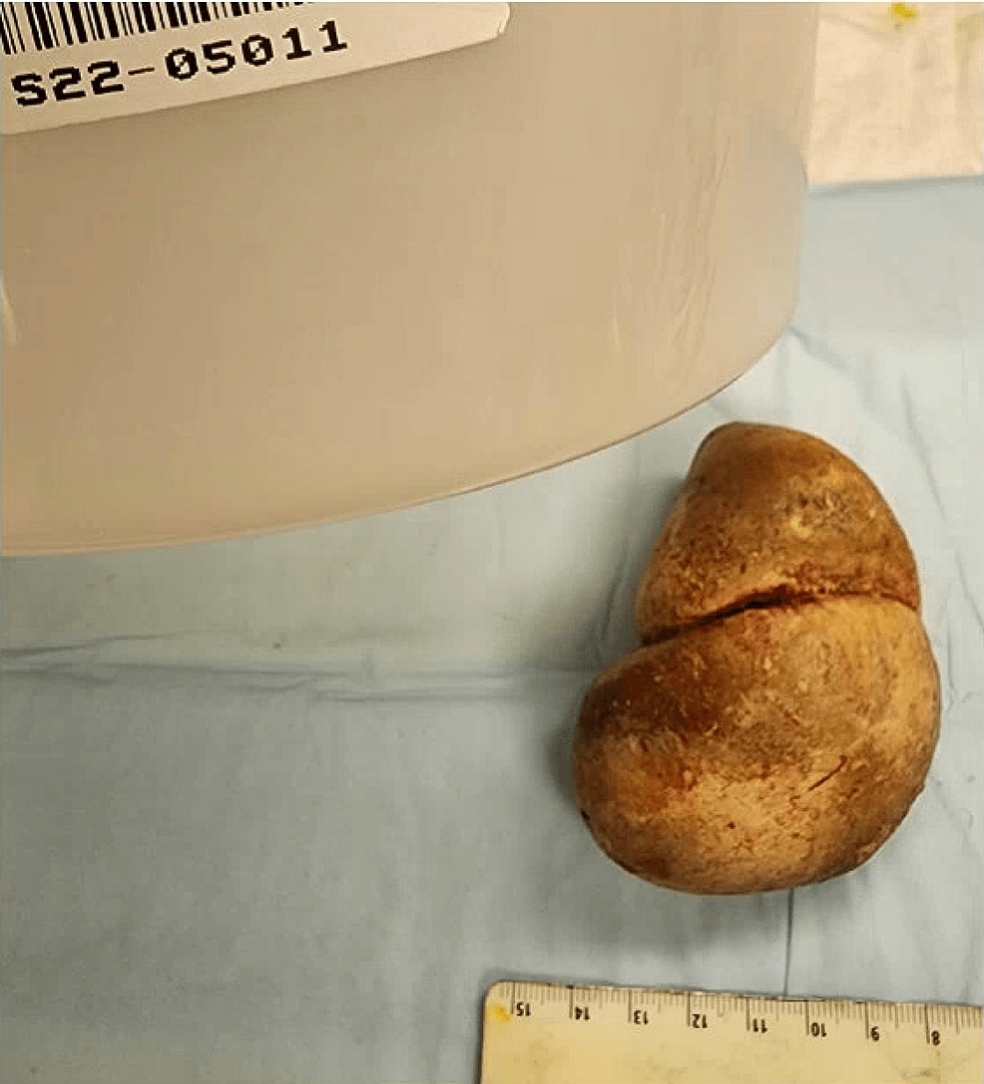
Gross image of 10 cm × 6 cm bladder stone after open cystolithotomy.

Computed tomography of the abdomen and pelvis without intravenous contrast demonstrated a bladder stone measuring 7.6 cm with bladder wall thickening, moderate-to-severe bilateral renal hydronephrosis, and hepatic cysts (Figures 1A, 1B). The patient was started on intravenous ceftriaxone 2 g daily for treatment of his urinary tract infection and the urological surgeon was consulted who recommended starting oxybutynin 5 mg three times daily. The next day, the patient’s urine output increased to 3.4 L. The patient’s urine culture grew extended-spectrum beta-lactamase-producing *Klebsiella pneumoniae*, and his antibiotics were immediately transitioned to intravenous meropenem 1 g every eight hours for a five-day course. On hospital day eight, the patient underwent an open cystolithotomy with the retrieval of a 10 × 6 cm bladder stone weighing 210 g and placement of a new 22 French urinary catheter (Figure 2). Microscopic analysis of the stone demonstrated a 100% composition of carbonate apatite. The patient was discharged with close follow-up with a primary care provider and a urologist.

## Discussion

Bladder stones do not typically cause bilateral hydronephrosis and post-obstructive acute kidney injury because they are not usually large enough to do so [5]. However, in the presence of a giant bladder stone, bladder outlet obstruction may ensue, leading to renal failure and hydronephrosis, which was demonstrated in our case and is consistent with the findings of several other case reports [1,2,5,6]. The standard therapy for bladder stones is transurethral lithotripsy; however, if the bladder stone size is very large, open suprapubic surgery is preferred, as these stones are not typically amenable to endoscopic therapies [2,4]. In our case, due to the large size of the bladder stone noted on computed tomography, the decision was made to perform an open cystolithotomy, which is supported by the management of other case reports [1,2,6]. Additionally, this is a Grade C recommendation from the American Urological Association and helped guide our management [7]. Open cystolithotomy allowed for retrieval of the intact stone and led to the resolution of the patient’s post-obstructive renal failure.

In our patient, the stone analysis revealed a 100% composition of carbonate apatite, which is a type of calcium phosphate. Calcium oxalate and calcium phosphate bladder stones are typically a result of kidney stones that pass into the bladder and become entrapped there, developing additional layers of stone deposition [4]. Consequently, we believe that the etiology of our patient’s carbonate apatite stone formation was likely related to his history of recurrent nephrolithiasis. Several studies have also noted that the male sex is a risk factor for developing bladder stones, and we believe this may have increased our patient’s risk for stone formation [3,5,6,8]. Additionally, our laboratory investigations identified vitamin D deficiency and elevated parathyroid hormone levels, indicating underlying secondary hyperparathyroidism. We believe that our patient had a higher propensity for developing calcium stones due to this condition, and it is likely the cause of his history of recurrent nephrolithiasis. Ultimately, the patient was started on vitamin D supplementation, and on follow-up, he has not had a recurrence of urolithiasis.

This case is unique for several reasons: (1) post-obstructive acute kidney injury related to bladder stones has seldom been reported in the literature, (2) secondary hyperparathyroidism due to vitamin D deficiency was likely associated with an increased risk of carbonate apatite stone formation, and (3) giant bladder stones are a rare entity in the current body of literature. In our experience, early diagnosis and prompt surgical management of the giant bladder stone led to a swift resolution of the patient’s medical ailments. Future studies should aim to assess the risk factors for developing giant bladder stones and the possibility of endourological interventions for the management of giant bladder stones.

## Conclusions

Giant bladder stones are a rare entity, and their association with post-obstructive acute kidney injury and bilateral hydronephrosis is even rarer. In this case, a 10 cm × 6 cm carbonate apatite stone was retrieved intact from the patient’s bladder, leading to the resolution of the patient’s acute kidney injury and bladder outlet obstruction. Based on our experience, we recommend prompt urological surgery evaluation in patients presenting with these clinical manifestations, as surgical intervention may be curative. Future studies may evaluate the role of endourological interventions for giant bladder stones and analyze specific risk factors related to the development of giant bladder stones.
